# Nanoclay Reinforced Ternary Blends Based on Biodegradable Polymers for Drug Delivery Application

**DOI:** 10.1155/2022/6585305

**Published:** 2022-09-07

**Authors:** Mohsin Ali, Sadullah Mir, Obaid-Ur-Rahman Abid, Mirza Arfan Yawer, Ihsan Ullah

**Affiliations:** ^1^Department of Chemistry, COMSATS University Islamabad, Abbottabad Campus, Abbottabad, Pakistan; ^2^Department of Chemistry, Hazara University Mansehra, Mansehra, Pakistan; ^3^Department of Chemistry, University of Education Lahore, Lahore, Pakistan; ^4^Institute of Chemical Sciences, University of Swat, Charbagh, Pakistan

## Abstract

In this study, ternary blends based on chitosan, polyvinyl alcohol, and polyethylene glycol reinforced with organically modified montmorillonite (nanoclay) clay were synthesized. These ternary blends were evaluated as transdermal drug delivery patches using tramadol as a model drug. The FTIR study showed interaction among important functional groups and compatibility among the mixing components. Among drug-loaded formulations, composite MA12 shows maximum thermal stability with 27.9% weight residue at 540°C. The prepared formulations exhibited crystalline nature as observed by XRD analysis. SEM studies revealed that there are no gaps and cracks in prepared films and nanoclay was found dispersed in the formulations. The swelling ratio was higher in pH 1.2 as compared to pH 4.5 and pH 6.8 buffers, and there was an increase in swelling with an increase in PVA concentration. Moreover, the drug release test performed in phosphate buffer pH 6.8 showed that tramadol release from nanocomposite films increases with an increase in PEG concentration. Permeation studies indicated that the rate of permeation increased with a decrease in PVA concentration. The permeation rate was found to be higher for samples without nanoclay. The overall results suggest nanocomposite films as excellent candidates for transdermal drug delivery application.

## 1. Introduction

The problems associated with other drug delivery methods can be overcome by using the transdermal drug delivery system (TDDS). The conventional drug delivery system mostly used is the oral route for which tablets or syrups are prepared. In this system, the drug passes through the stomach, liver, and kidneys. During this process, these organs are badly affected by the drug. Also, the effective drug content is very low in this case. Therefore, the transdermal drug delivery system is used to overcome these problems. It provides an alternate route to bypass the stomach, liver, and kidneys and gives both systemic and local therapeutic effects. The retention time of drugs in the body is also short in the conventional system, and for patients suffering from paralyzes or nerve pain, an attendant is usually required. In TDDS, the patch containing the drug is applied on the body which releases the drug slowly and helps to reduce the job of the attendant. The issues like overdosing and underdosing can be controlled with TDDS [1]. The transdermal drug delivery system makes use of transdermal patches which help to release the drug over an extended time, thus avoiding frequent dosing. They have lesser side effects as compared to conventional dosage forms [2].

Tramadol HCl is an analgesic that helps to relieve anxiety and depression. It exhibits both opioid and nonopioid characteristics. Apart from these advantages, some side effects are attributed to immediate excretion and fast metabolism. Therefore, a controlled delivery system is required to cope with the problem of multiple dosing [3]. To formulate systems for controlled drug release, biodegradable polymers are used. Common biodegradable polymers are polyethylene glycol (PEG), chitosan (CS), poly-*ε*-caprolactone (PCL), soy protein, and copolymers of polyglycolide, polylactic acid (PLA), poly-3-hydroxybutyrate, polyglycolic acid (PGA), and alginate [4]. Chitosan is the second most abundantly found polysaccharide in nature and is used to design systems for drug delivery [5]. Chitosan (CS) possesses much importance because of its nontoxicity, biocompatibility, and biodegradability [6], and it can be synthesized from chitin [7]. PVA is another important biopolymer and because of its chemical resistance, low protein adsorption property, biocompatibility, and good water solubility, it is widely used for advanced biomedical applications like artificial organs, contact lenses, wound dressings, wound healing [8], and drug delivery systems [9, 10]. PVA is also noncytotoxic [11]. Polyethylene glycol (PEG) is used to formulate controlled drug release systems as it is biodegradable, biocompatible, and safe for use [12, 13]. As CS, PVA, and PEG are biodegradable and biocompatible and their ternary blends produce strong interaction among their functional groups which extends the drug release time, they make a desirable system for controlled drug release. Chitosan, PVA, and PEG have been used by several researchers for the preparation of polymer nanocomposites (PNCs) [12, 14].

Clay-reinforced polymer nanocomposite is obtained by the combination of organic polymer matrices and organophilic clay nanofillers [15]. The blend properties are enhanced by adding nanofillers making it compatible with biological systems [16, 17]. When the polymers and clay combine at the atomic level, this makes the basis for a significant class of organic-inorganic nanocomposites [18]. Montmorillonite (MMT) is a hydrated aluminosilicate clay mineral having a platelet-like structure [19]. On the surface of clay, reactive species are present that interact with the drug and the polymers through an ion exchange mechanism (intercalation and exfoliation). MMT is found to contribute to controlled drug release [20].

Drug molecules get transported through the skin in two steps i.e., first, the drug gets diffused into deeper tissues after crossing the stratum corneum. In the second step, it reaches the targeted area through the blood plasma and performs its required function. The rate and extent of drug transported vary with ionic strength, size, H-bonding, log *p* value, and physicochemical properties [21]. The issues found with conventional systems, such as nonuniform dosing concentrations and bad effects on the liver and fast excretion and fast metabolism of tramadol, can be encountered by making transdermal nanocomposite patches of tramadol. Hydrogel scaffolds are also used in drug delivery nowadays, but we preferred to use thin films for this purpose as sometimes there are issues with the hydrogels, such as nonbiodegradability, nonbiocompatibility, burst drug release during swelling, fast release from large porous hydrogels, drug deactivation, the toxicity of residual small molecule crosslinkers, and low mechanical strength [22].

In the present work, novel tramadol formulations in the form of nanocomposite films for controlled release having minimal side effects are reported. These nanocomposites having negligible side effects may be of much importance to the pharmaceutical industry. Research work aims to find out the role of CS-PVA-PEG nanocomposite thin films in the controlled delivery of tramadol. To assess the structure, thermal properties, and morphology of nanocomposite films, Fourier transform infrared spectroscopy, thermogravimetric analysis, X-ray diffraction, and scanning electron microscopy were used. Pharmaceutical tests such as swelling and permeation through rat skin by employing Franz diffusion cell, erosion studies, drug content uniformity studies, water content, and dissolution studies were also carried out to evaluate the control drug delivery system.

## 2. Methods

### 2.1. Materials

Chemicals were obtained from different suppliers in pure or distilled form. Chitosan, PVA, PEG, acetic acid, glycerol, KCl, NaOH, and nanoclay were supplied by Sigma-Aldrich. NaOAc and KH_2_PO_4_ were obtained from Merck (Germany) and Daejung (South Korea), respectively. Tramadol was a gift from Global Pharmaceuticals (Islamabad, Pakistan), and distilled water was obtained from COMSATS University Abbottabad campus.

### 2.2. Synthesis of CS-PVA-PEG Thin Films

The solvent casting technique was used with some variations in the previously reported method for the preparation of CS-PVA-PEG nanocomposites [13]. Chitosan, PVA, and glycerol (plasticizer) were added with constant stirring to the previously dissolved 1% aqueous acetic acid solution of PEG. Tramadol was added after 15 minutes, followed by the addition of nanoclay to the polymer mixture with constant stirring. A clear solution was obtained after stirring the mixture at 60°C for half an hour. It was transferred to Petri dishes after complete dissolution and kept for 24 h in an oven at 50°C until completely dried. Thin films were obtained upon drying. By varying the quantity of PVA, PEG, nanoclay, and tramadol, 12 different formulations were obtained as shown in Table 1.

### 2.3. Characterization Techniques

The synthesized formulations were characterized by the following analytical techniques.

#### 2.3.1. Fourier Transform Infrared Analysis

For the structure determination of prepared formulations, Fourier transform infrared spectroscopy (Thermo Scientific Nicolet 6700″USA) was used. The prepared films were grinded and mixed with KBr. The spectrum was scanned between 4000 and 500 cm^−1^ [23].

#### 2.3.2. XRD Analysis

X-ray diffraction analysis helped in the determination of the amorphous or crystalline nature of the prepared nanocomposites. The samples were analyzed on the Philips XPERT PRO 3040/60 X-ray diffractometer over a 2*θ* range of 5–90° [24].

#### 2.3.3. Thermal Analysis

The Shimadzu DTG-60H NG12 5AW Thermal Analyzer (Nottinghamshire, the United Kingdom) was used to analyze the thermal stability of the prepared films. Samples were placed in an analytical pan under the N_2_ atmosphere (flow rate approx. 20 mL/min). The samples were left for thermal decomposition at 0–600°C after positioning approximately 4 mg of the sample in an aluminum pan to continuously analyze the weight loss at increasing temperatures [25].

#### 2.3.4. Energy Dispersive X-Ray Analysis

To perform EDX analysis, the (JSM 6400F SEM; Jeol) scanning electron microscope was used. The gold coating of prepared samples was carried out on an aluminum holder. The EDX analysis was also carried out at 20.194 kV. Energy dispersive X-ray helped to determine the elemental composition and purity of the mixing components [24].

#### 2.3.5. Scanning Electron Microscopic Analysis

To study the morphological details of the prepared formulations, the JSM 6400F scanning electron microscope was used. The voltage was set to 5–15 kV. The gold coating was carried out on an aluminum holder [26].

#### 2.3.6. Solubility Study of Tramadol HCl

Different solvents were used to perform solubility studies. Tramadol was dissolved in 50 mL of different solvents. The solutions underwent stirring at 37 ± 0.5°C for 24 hours, and at the end, they were centrifuged for the removal of the extra drug. After filtration, proper dilution of the supernatant layer was performed with respective solvents and tramadol concentration was measured at 218 nm [27].

#### 2.3.7. Calibration Curve

For the estimation of tramadol concentration, a standard calibration curve was plotted. To prepare the stock solution, tramadol (100 mg) was dissolved in KH_2_PO_4_, pH 6.8buffer (100 ml). Nine different solutions were obtained by diluting the tramadol solution in a range of 2–20 *μ*g/ml. These solutions were analyzed at 218 nm with phosphate buffer (pH 6.8) as a reference [28].

#### 2.3.8. Drug Content Uniformity Test

In a 100 mL volumetric flask, 30 mg of sample was dissolved in phosphate buffer with pH 6.8 and volume made up to the mark. The sample solution underwent stirring for 24 hours. The sample aliquots were collected after 24 hours and diluted with the same buffer for UV absorption measurement at 218 nm [28].

#### 2.3.9. Swelling Studies

HCl (pH 1.2), NaOAc (pH 4.5), and phosphate (pH 6.8) buffer solutions were used for swelling studies. 30 mg of the sample was dissolved in 30 ml of buffers separately. The samples were taken out at an interval of 1, 2, 3, 4, 5, and 6 hours, the extra buffer was removed with tissue paper, and their weights were taken. Using the following equations, the swelling ratio (SR) and percent water content (%) were measured [29]:

(1) $$\begin{array}{c} \text{SR}=\frac{W_{s}}{W_{d}},\\ \text{Percent water content}\left(\%\right)=\left[\frac{W_{s}-W_{d}}{W_{s}}\right]\times 100, \end{array}$$

where *W*_*d*_ and *W*_*s*_ show the weights of dry and swollen films, respectively.

#### 2.3.10. Erosion Analysis

Wet samples from the swelling experiments were oven-dried for 20 minutes at 50°C and weighed at time intervals of 1, 2, 3, 4, 5, and 6 hours until a constant weight was obtained. A triplicate experiment was performed to estimate the percent film erosion (%) by using the following equation [30]:

(2) $$\begin{array}{c} \text{Film erosion}\left(\%\right)=\left[\frac{W_{0}-W_{2}}{W_{0}}\right]\times 100, \end{array}$$

where *W*_0_ and *W*_2_ are the weights of wet and dry films, respectively.

#### 2.3.11. Preparation of Rat Skin

The Department of Pharmacy, COMSATS University Islamabad, Abbottabad, Pakistan, supplied eighteen Sprague-Dawley rats having an average weight of 200–250 g. Rats were kept in alternating light and dark cycles, and the standard procedure was followed in this experiment [31]. Chloroform was used for anesthesia. Electrical and hand blade were used to shave the belly skin, and then, the skin was removed. It was followed by cleansing dermal fat and placement of the skin in 0.9% NaCl solution for the removal of enzymes and debris. The skin was folded in an aluminum sheet after washing with disinfected water and kept at 20°C for use. Frozen excised rat skin was taken out of the freezer before the experiment, and it was adjusted between the compartments of the Franz diffusion cells with the stratum corneum side facing the donor compartment and the dermal side facing the receptor compartment [21].

#### 2.3.12. Permeation Analysis

Permeation study was carried out by using Franz diffusion cells [32]. The skin was fixed between the compartments of diffusion cells which were held together by clamps and the receptor part was filled with phosphate buffer, pH 6.8, so that the buffer solution touched the rat skin. Weighed sample (40000 *μ*g) having 2250 *μ*g of the drug was used in the experiment. The sample was placed on the rat skin and protected with an aluminum covering to avoid drying. The temperature was kept at 37 ± 0.5°C, and readings were noted down at an interval of 1, 2, 3, 4, 5, 6, and 24 hours in the form of small aliquots of sample collected from the receptor compartment and then replaced with the same amount of buffer. Samples were analyzed under a UV spectrophotometer at 218 nm for tramadol concentration measurement.

#### 2.3.13. Dissolution Study

The drug release experiment was carried out with slight modifications to a previously used method by Rasool et al. [33]. 100 mg of the nanocomposite film was dissolved in 250 milliliters of KH_2_PO_4_ buffer, pH 6.8, in a beaker, at a 100 rpm magnetic bar stirring rate for 12 hours with the temperature kept at 37 ± 0.5°C. Moreover, a 5 mL sample was collected at time intervals of 1, 2, 3, 4, 5, 6, and 12 hours for analysis. The dissolution medium was substituted with the same quantity of fresh buffer. The experiment was performed in triplicate. The collected samples were analyzed at 218 nm under a UV spectrophotometer to estimate the percent drug release.

#### 2.3.14. Statistical Analysis

GraphPad Prism version 9.4.0 for Windows (GraphPad Software, San Diego, California USA (https://www.graphpad.com/)) was used for statistical analysis in this study.

## 3. Results and Discussion

### 3.1. Fourier Transform Infrared Analysis

**Figure 1:**
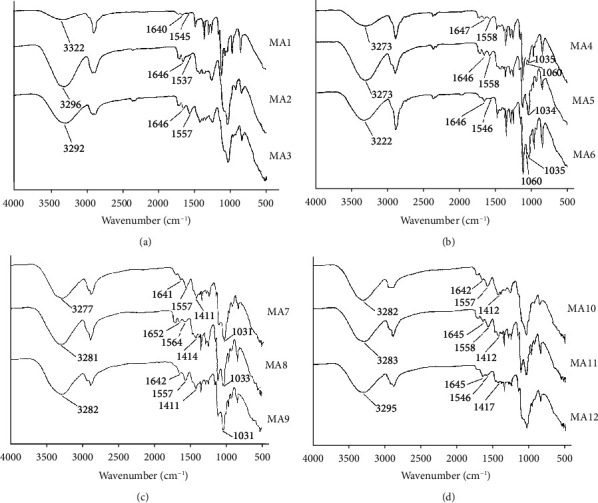
FTIR spectra of chitosan-PVA-PEG nanocomposite films. (a) MA1–MA3. (b) MA4–MA6. (c) MA7–MA9. (d) MA10–MA12.

FTIR analysis shows different vibrational modes for N-H, C=O, OH, Si-O-Si, and C-N in synthesized nanocomposites as shown in Figure 1 and Tables S1–S4 (supplementary data). The OH group in pure chitosan, PVA, and PEG appears at 3334, 3550, and 3441 cm^−1^, respectively [34–37]. The OH stretching frequencies in MA1, MA2, and MA3 appear at 3322, 3296, and 3292 cm^−1^, respectively, as shown in Figure 1(a). A lowering in wavenumber can be seen when compared to the reported work. This lowering of the OH frequencies is due to the compatibility between the mixing polymers as the energy is lowered after bond formation. Here, intermolecular and intramolecular H-bonding causes a lowering in the OH frequencies. As shown in Figure 1(d), the OH group in drug-loaded samples MA10, MA11, and MA12 appears at 3282, 3283, and 3295 cm^−1^, respectively, showing the same lowering of wavenumber trend as observed in MA1-MA3. The amide C=O group of chitosan appears at 1648 cm^−1^ [38]. However, the amide group in MA1, MA2, and MA3 appears at 1640, 1646, and 1646 cm^−1^, respectively, as shown in Figure 1(a). The C=O in drug-loaded samples MA10, MA11, and MA12 appear at 1642, 1645, and 1645 cm^−1^, respectively, as shown in Figure 1(d). The N-H group of pure chitosan appears at 1594 cm^−1^ [38]. For nondrug-loaded samples MA1, MA2, and MA3 appear at 1545, 1537, and 1557 cm^−1^, respectively. In the drug-loaded samples MA10, MA11, and MA12, the NH group appears at 1557, 1558, and 1546 cm^−1^, respectively. A lowering in wavenumber is also observed for C=O and NH groups in prepared composites as compared to pure chitosan which is also due to H-bonding which accounts for efficient mixing of the polymers and their compatibility. The OH group in nondrug-loaded nanoclay-containing samples MA4, MA5, and MA6 appears at 3273, 3273, and 3222 cm^−1^, respectively, as shown in Figure 1(b), showing the same trend as observed for MA1–MA3 (Figure 1(a)) and MA7–MA9 (Figure 1(c)).

When comparing the Si-O-Si stretching vibrations of the prepared formulations with the reported work, it is found that for pure nanoclay, it is in the range of 1068–1000 cm^−1^ [39]. Similar results are reported in our study. For instance, Si-O-Si in the nondrug-loaded nanocomposites MA4, MA5, and MA6 appear at 1060/1035, 1034, and 1060/1035 cm^−1^, respectively. However, in the drug-loaded nanocomposites, MA7, MA8, and MA9 (Si-O-Si) groups appear at 1031, 1033, and 1031 cm^−1^, respectively, as shown in Figure 1(c).

Literature reports that the C-N group of pure tramadol appears at 1439 cm^−1^ [40]. For drug-loaded nanocomposites MA7, MA8, and MA9, it appears at 1411, 1414, and 1411 cm^−1^, respectively as shown in Figure 1(c). For drug-loaded composites, MA10, MA11, and MA12, the C-N group appears at 1412, 1412, and 1417 cm^−1^, respectively, as shown in Figure 1(d). A comparison of our formulations with the literature shows a lowering of wavenumber for the C-N group of the prepared formulations. This lowering in wavenumber is also due to the H-bonding which leads to increased compatibility between mixing components.

### 3.2. Thermogravimetric Analysis

**Figure 2:**
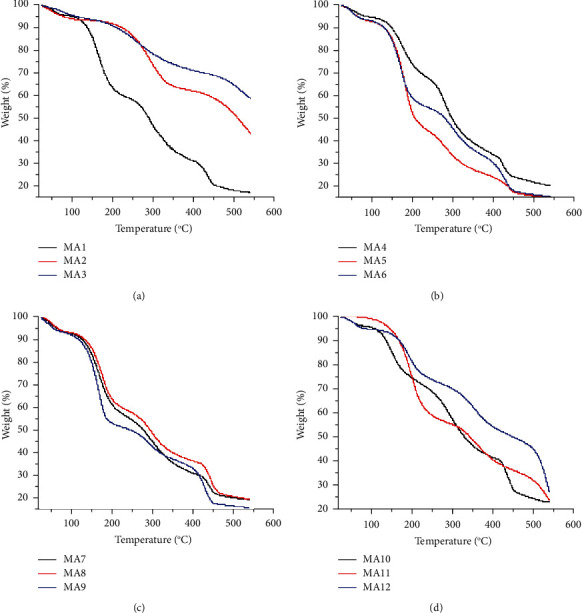
TGA thermograms of chitosan-PVA-PEG nanocomposite films: (a) MA1–MA3, (b) MA4–MA6, (c) MA7–MA9, and (d) MA10–MA12.

The thermogravimetric analysis shows the thermal stabilities of samples over variable temperature ranges. The results are shown in Tables S5–S8 (supplementary data). The samples MA1–MA3 show three stages of degradation ranging from 0 to 132°C, 132–209°C, and 209–404°C as shown in Figure 2(a). The sample MA3 shows maximum thermal stability with 28.5% weight loss at 404°C. MA2 composition also exhibits considerable thermal stability at 540°C with a residual weight of 43.9%. However, MA1 (Figure 1(a)) shows the least thermal stability having a weight residue of 17.3% at 540°C.

Among clay dispersed samples MA4–MA6 (Figure 1(b)), sample MA4 is most stable with 56.6% weight loss at 327°C and a weight residue of 20.6% at 540°C. MA5 and MA6 have almost the same thermal stability with weight loss of 70% and 59.6% at 327°C and weight residues of 15.6% and 15.9%, respectively, at 540°C as shown in Figure 2(b).

Drug-loaded nanocomposites MA7–MA9 have thermal stability comparable to that of nondrug-loaded nanocomposites MA4–MA6. Among these, the formulation MA9 shows minimum stability with 16.2% weight residue at 540°C, while MA7 and MA8 have almost the same thermal stability with 19.6% and 19.7% weight residues at 540°C as shown in Figure 2(c).

The drug-loaded samples without clay, i.e., formulations MA10–MA12 have higher thermal stability when compared to clay-containing drug-loaded samples (MA7–MA9). The highest thermal stability is shown by MA12 (Figure 2(d)) with 34.5% weight loss at 340°C and a weight residue of 27.9% at 540°C. The maximum weight loss of 51.3% is shown by MA10 (Figure 2(d)) at 340°C with a weight residue of 23.4% at 540°C. When comparing the drug-loaded composites MA10–MA12 with nondrug-loaded composites MA1–MA3, it is obvious that nondrug-loaded samples have very high thermal stability compared to drug-containing composites. A comparison between clay-containing formulations MA4–MA6 and nonclay formulations MA1–MA3 shows that samples without clay have greater thermal stability compared to clay-containing formulations. Overall comparison of formulations shows that polymer composites without clay and drug (MA1–MA3) have maximum thermal stability compared to nondrug-loaded clay-containing nanocomposites (MA7–MA9) and drug-loaded composites without clay (MA10–MA12). The maximum stability is shown by MA3 with the maximum concentration of PEG. A look at MA1–MA3 shows an increase in thermal stability with an increase in PEG concentration.

A comparison can be easily made between our study and the reported work. Falqi et al. reported the thermal stability enhancement of PVA/PEG/graphene with the increase in PEG concentration. The comparison shows that there are different steps in the TGA curves. The first curve appears at around 90°C where physisorbed water was lost [41]. Pure PVA shows major degradation in the temperature range of 243–387°C as reported by Jose et al. [42]. Literature reports that the thermal decomposition of PEG begins above 330°C, and PEG is thermally more stable as compared to PVA [43, 44]. Our work shows that the maximum thermal decomposition of the samples occurs in a temperature range of 200–400°C, and the thermal stability increases with an increase in PEG concentration.

### 3.3. X-Ray Diffraction Analysis

**Figure 3:**
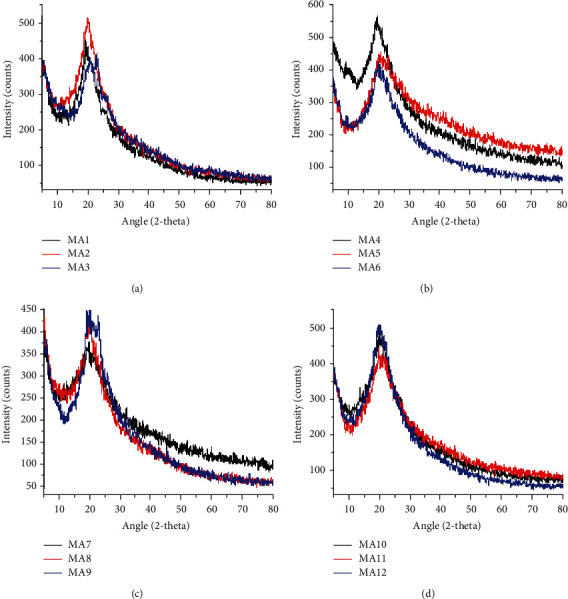
XRD patterns of chitosan-PVA-PEG nanocomposite films: (a) MA1–MA3, (b) MA4–MA6, (c) MA7–MA9, and (d) MA10–MA12.

The prepared formulations contain three polymers (chitosan, PVA, and PEG), nanoclay, and a drug tramadol) as shown in Figure 3 and Table S9 (supplementary data). Feng et al. reported that chitosan appears at 2*θ* = 10.6°, 11.4°, 20.1°, and 20.4° [45]. Ricciardi et al. reported that PVA appears at 2*θ* = 19.4° and 20° [46], while PEG was reported at 2*θ* = 19.23° and 23.34° by Ahmad et al. [47]. When comparing the prepared formulations with the reported values, it is found that all three polymers appear around 20°, so it is difficult to differentiate the peaks of chitosan and PVA from each other. However, chitosan can be differentiated by its peak around 10°. PEG can be traced at 23.34° as shown in Figure 3. The study shows that these polymers are present in crystalline form as intense sharp peaks are reported around 20°.

Samples MA4–MA6 contain nanoclay dispersed in polymers. Literature shows that nanoclay appears at 2*θ* = 6.22° [48]. When we compare our results with the literature, the presence of nanoclay in crystalline form can be confirmed by the sharp peaks around 5° and 6° as shown in Figure 3. Samples MA7–MA9 contain both clay and drug (tramadol) dispersed in polymers (Figure 3(c)). Literature shows that tramadol appears at 2*θ* = 10°, 12°, 16°, 18°, 24°, and 26° as reported in a study by Sohail et al. [49]. A comparison with the literature can be made and the presence of tramadol in crystalline form is confirmed by the intense sharp peaks around 2*θ* = 18° and 26° as shown in Figure 3. XRD analysis shows that all of the components are present in crystalline form. A shift in peak value from 19° to 21° can be seen in composites containing polymers only (MA1–MA3) which shows enhancement in the crystallinity of these polymers. This upshift can also be observed in nanoclay and tramadol-containing samples, i.e., both nanoclay and tramadol enhance the crystalline behavior of polymers and show excellent compatibility (Figure 3).

### 3.4. Scanning Electron Microscopy

**Figure 4:**
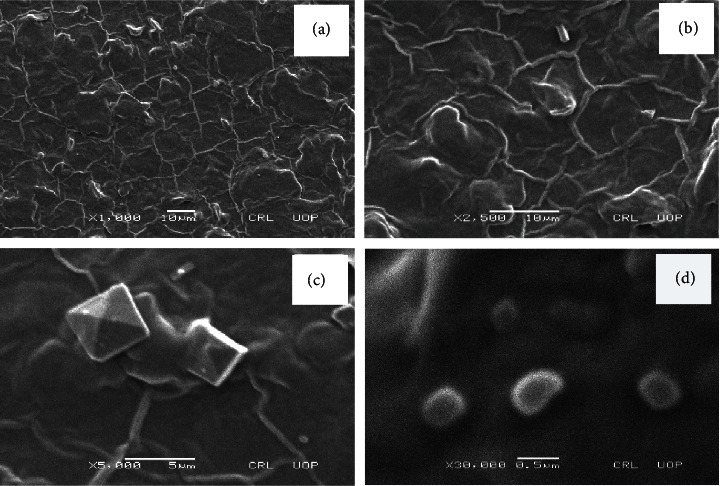
Scanning electron micrographs of MA4 at (a) x1000, (b) x2500, (c) x5000, and (d) x30000 magnifications.

**Figure 5:**
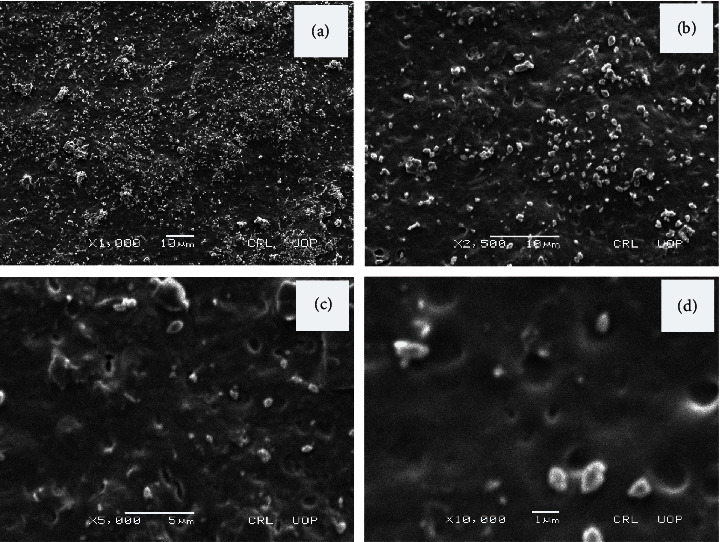
SEM micrographs of MA8 at (a) x1000, (b) x2500, (c) x5000, and (d) x10000 magnifications.

SEM images revealed that the nanoclay was evenly distributed in the matrix as shown in Figures 4 and 5. There were no gaps and cracks in the prepared films. Nanoclay is compatible with the polymer matrix. The clay particles appear in the form of small spots in high-resolution images. The particle size of nanoclay was observed in the range of 300–500 nm. Large-sized integrated clay bundles can be seen which are attributed to the presence of nondispersed clay particles. The agglomeration results from the bonding interactions among MMT particles [50].

When we compare with the literature, it is found that these agglomerates were also reported by Alekseeva et al. in their study on montmorillonite/ionic liquid composites [51].

### 3.5. Energy Dispersive X-Ray Analysis

**Figure 6:**
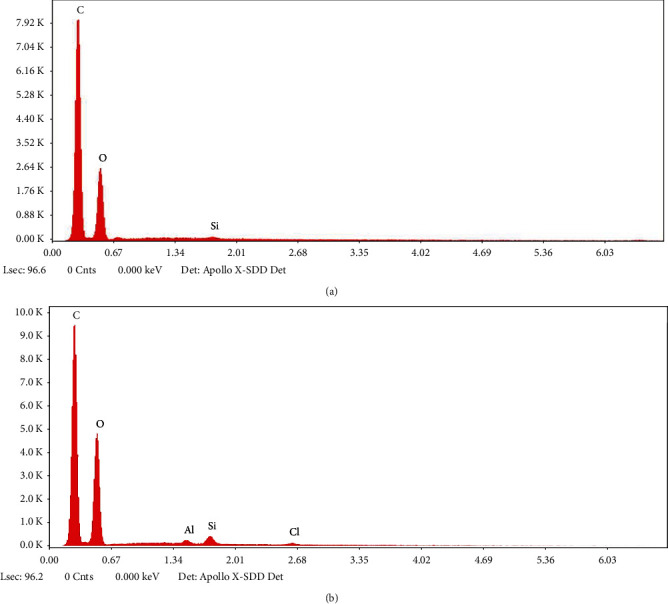
EDX profile of chitosan-PVA-PEG nanocomposite films: (a) MA4 and (b) MA8.

The elemental composition of selected formulations MA4 (Figure 6(a)) and MA8 (Figure 6(b)) was studied by EDX analysis. It helped to determine the purity of mixing components. The peaks for O and C are intense showing a greater proportion of the chitosan, PVA, and PEG polymers. Aluminum and silicon peaks are attributed to nanoclay. The chlorine peak confirms the presence of tramadol hydrochloride [24].

### 3.6. Calibration Curve Plot

**Figure 7:**
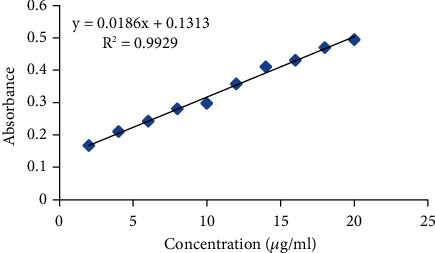
Calibration curve of tramadol.

The standard calibration curve of tramadol was plotted using a series of dilutions as shown in Figure 7. These dilutions were made in KH_2_PO_4_, pH 6.8, buffer in a 2–20 µg/ml range. The *Y*-equation appeared to be 0.0186x + 0.1313, and the *R*^2^ (coefficient of determination) value was found to be 0.9929 [24].

### 3.7. Swelling Analysis

**Figure 8:**
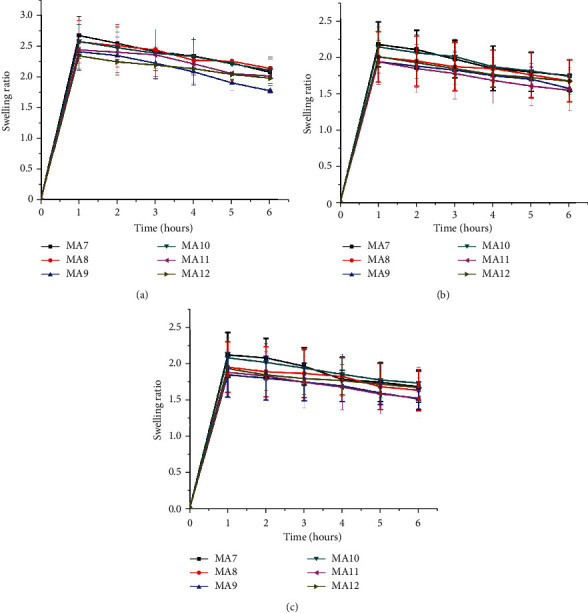
The swelling ratio for CS-PVA-PEG nanocomposite formulations in (a) hydrochloric acid-pH 1.2, (b) sodium acetate-pH 4.5, and (c) phosphate-pH 6.8 buffer solutions.

The drug-containing samples underwent swelling analysis in three different buffers, i.e., HCl, pH 1.2; NaOAc, pH 4.5; and phosphate, pH 6.8 buffer solutions as shown in Figure 8. The swelling was found to be higher in HCl buffer as compared to NaOAc and phosphate buffers. This phenomenon was explained by Abdelaal et al. in their study on chitosan/PVA blends. It is since in a more acidic environment, OH and NH_2_ groups of chitosan get protonated and these protonated groups, in turn, provide sites to H_2_O molecules for solvation.

Also, there is an increased swelling with increased PVA concentration because PVA is hydrophilic and thus enhances the swelling capacity of prepared films. In pH 1.2 buffer, the swelling ratio (2.67 ± 0.31) was maximum for MA7 with PVA : PEG of 75 : 25, while minimum swelling ratio (2.34 ± 0.22) was found for MA12 with PVA : PEG of 25 : 75. When we compare with the literature, it can be seen that our results were consistent with the findings of Abdelaal et al. [52]. In their study on chitosan/PVA blends, the swelling percent of chitosan was 270%, while it increased to 300%, 340%, and 360% upon blending with 50%, 60%, and 75% PVA, respectively.

### 3.8. Erosion Studies

The erosion studies were also carried out for drug-containing samples in HCl, NaOAc, and phosphate buffers having pHs of 1.2, 4.5, and 6.8, respectively, as shown in Table 2. The results show that erosion is maximum in pH 1.2 buffer. The reason behind this fact is that the pH 1.2 buffer has maximum swelling and samples have maximum buffer content in this case as explained above (swelling study). These swollen samples are taken out of the buffer solutions and left for drying. The samples now undergo erosion, i.e., loss of sample contents with buffer loss. The samples having maximum swelling (in pH 1.2 buffer) will have maximum erosion. Our study shows that erosion also varies with variation in PVA : PEG ratio. Erosion increases with an increase in PEG concentration and decrease in PVA concentration. When comparing the literature, we found that similar findings were reported by Gilani et al. in their work on chitosan/PEG nanocomposites [24]. Gilani et al. reported that the sample containing 75% PEG showed the maximum weight loss (76.2 ± 0.56%), whereas the sample containing 0% PEG showed the minimum weight loss (36.6 ± 0.85%).

### 3.9. Dissolution

**Figure 9:**
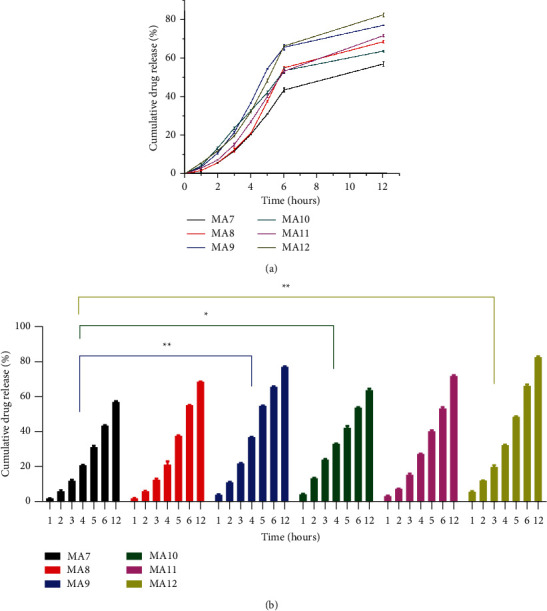
(a) Tramadol HCl in vitro releases comparison for formulations MA7-MA12. (b) Statistical analysis (*n* = 3) using Dunnet's multiple comparisons test (*p* values: ^*∗*^*p* < 0.05 and ^*∗∗*^*p* < 0.01).

The dissolution experiment was performed in triplicate using KH_2_PO_4_, pH 6.8, buffer to estimate the percent drug release in drug-containing samples as shown in Figure 9. The readings were taken at an interval of 1, 2, 3, 4, 5, 6, and 12 hours.

Among nanoclay-containing samples, MA7 with PVA : PEG of 75 : 25 has cumulative percent drug release (56.97 ± 0.00404%) and MA9 with PVA : PEG of 25 : 75 has cumulative percent drug release (77.04 ± 0.00115%). Among samples without nanoclay, MA10 with PVA : PEG of 75 : 25 has cumulative percent drug release (63.65 ± 0.00907%) and MA12 with PVA : PEG of 25 : 75 has cumulative percent drug release (82.35 ± 0.00755%). The drug release increases with an increase in PEG concentration or a decrease in PVA concentration. When comparing the literature, we found that similar results were reported by Gilani et al. where maximum cumulative percent drug release (35.51 ± 0.26117%) was reported for a sample containing 75% PEG, whereas minimum cumulative percent drug release (29.88 ± 0.29987%) was reported for a sample containing 0% PEG [24]. Thus, PVA helps to slow down drug release. The formulations having nanoclay, i.e., MA7, MA8, and MA9 have lower drug release compared to formulations without nanoclay, i.e., MA10, MA11, and MA12. Nanoclay also plays an important role in the controlled release of drugs. Nanoclay retards drug release [53].

### 3.10. Permeation

**Figure 10:**
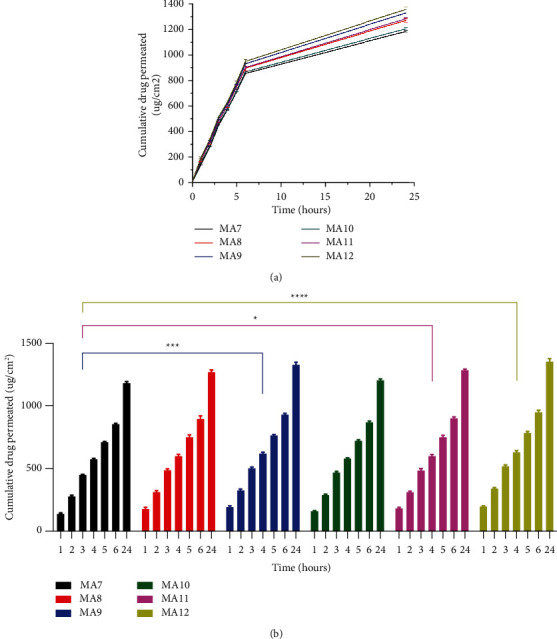
(a) Tramadol HCl Permeation and Comparison for Formulations MA7-MA12. (b) Statistical Analysis (*n* = 3) using Dunnet's Multiple Comparisons Test (*p* values: ^*∗*^*p* < 0.05*p*=0.0003 and *p* < 0.0001).

Permeation studies were performed for the drug-containing samples to estimate the rate of drug release through rat skin. The triplicate experiment was performed at a time interval of 1, 2, 3, 4, 5, 6, and 24 hours in each of the three buffer solutions i.e., hydrochloric acid (pH 1.2), sodium acetate (pH 4.5), and potassium phosphate (pH 6.8) buffer solutions as shown in Figure 10.

Among nanoclay-containing samples MA7 with PVA : PEG of 75 : 25 has cumulative drug permeation of 1183.34 ± 8.63 *μ*g/cm^2^ and MA9 with PVA : PEG of 25 : 75 has drug permeation of 1328.58 ± 18.25 *μ*g/cm^2^. Among samples without nanoclay, MA10 with PVA : PEG of 75 : 25 has cumulative drug permeation of 1204.11 ± 10.36 *μ*g/cm^2^ and MA12 with PVA : PEG of 25 : 75 has drug permeation of 1356.08 ± 20.83 *μ*g/cm^2^. The permeation results show that the rate of permeation increases with a decrease in PVA concentration (or an increase in PEG concentration). When we compare the literature, we come to know that our results were consistent with those reported by Gilani et al. [24]. The highest cumulative drug permeation (2405.15 ± 10.97 *μ*g/cm^2^) was reported for a sample containing 75% PEG, whereas a sample containing 0% PEG showed the lowest drug permeation (1576.85 ± 11.81 *μ*g/cm^2^).

The permeation is also found to be more for samples without nanoclay. Nanoclay-containing samples have a less permeation rate compared to those samples which do not contain nanoclay. Thus, nanoclay hinders drug release as shown in a previous study by Banik et al. [53].

### 3.11. Drug Content Uniformity

The prepared formulations containing the drug were tested for drug content uniformity. The sample patches were cut from the center and proximity. Triplicate experiment was performed for these two sets of patches in phosphate buffer having pH 6.8. Maximum drug loading was found for MA11 (95.89 ± 0.86)%, while minimum drug loading was observed for MA12 (91.51 ± 1.20)%. The experiment showed even distributions of the drug in all the samples, i.e., the contents were nearly the same in the center and proximity. The drug particles were distributed evenly throughout the prepared formulations [24].

## 4. Conclusion

The FTIR study showed interaction among important functional groups and compatibility in the mixing components. The FTIR analysis showed that there was a lowering of wavenumber for the composites compared to the pure polymers. This lowering was mainly due to H-bonding. The lowering of wavenumber refers to the strong bonding interactions between the mixing polymers.

Among drug-loaded formulations, composite MA12 shows maximum thermal stability with 27.9% weight residue at 540°C. Nondrug-loaded composite MA2 is the most stable of all the formulations with 43.9% weight residue at 540°C. It shows that the drug (tramadol HCl) does not have any role in enhancing the thermal stability of prepared formulations. The study reveals that the maximum thermal decomposition of the samples occurs in the 200–400°C temperature range and the thermal stability increases with an increase in PEG concentration. XRD analysis shows that these polymers are present in crystalline form as intense sharp peaks are reported around *2θ* = 20°. SEM studies revealed that there are no gaps and cracks in prepared films and nanoclay was found dispersed in the formulations. The particle size of nanoclay was observed in the range of 300–500 nm.

Drug release properties are considerably influenced by film composition. The dissolution, swelling, erosion, and permeation rates can be altered by varying PVA: PEG ratio in nanocomposite films. The swelling increases by increasing polyvinyl alcohol concentration or decrease in polyethylene glycol concentration. In pH 1.2 buffer, the swelling ratio (2.67 ± 0.31) was maximum for MA7 with PVA: PEG of 75 : 25, while minimum swelling ratio (2.34 ± 0.22) was found for MA12 with PVA: PEG of 25 : 75. However, erosion, drug release, and permeation rate decrease with an increase in polyvinyl alcohol concentration.

Among nanoclay-containing samples MA7 with PVA: PEG of 75 : 25 has cumulative percent drug release of 56.97 ± 0.00404% and MA9 with PVA: PEG of 25 : 75 has cumulative percent drug release of 77.04 ± 0.00115%. Among samples without nanoclay, MA10 with PVA: PEG of 75 : 25 has cumulative percent drug release of 63.65 ± 0.00907% and MA12 with PVA : PEG of 25 : 75 has cumulative percent drug release of 82.35 ± 0.00755%. Nanoclay also serves to control the rate of drug release, i.e., the higher the concentration of nanoclay in the nanocomposite films, the lower the rate of drug release. Among nanoclay-containing samples, MA7 with PVA: PEG of 75 : 25 has cumulative drug permeation of 1183.34 ± 8.63 *μ*g/cm^2^ and MA9 with PVA : PEG of 25 : 75 has a drug permeation of 1328.58 ± 18.25 *μ*g/cm^2^. Among samples without nanoclay, MA10 with PVA : PEG of 75 : 25 has cumulative drug permeation of 1204.11 ± 10.36 *μ*g/cm^2^ and MA12 with PVA : PEG of 25 : 75 has a drug permeation of 1356.08 ± 20.83 *μ*g/cm^2^. The permeation results show that the rate of permeation increases with a decrease in PVA concentration (or an increase in PEG concentration).

Based on their properties, the prepared nanocomposites could serve as potential materials for transdermal drug delivery. Biocompatibility and cytotoxicity of the thin films were not investigated this time, but they will be the focus of our future study on these nanocomposite thin films. With these studies, it will be easier to define their role in drug delivery applications.

## Figures and Tables

**Table 1:** Composition of chitosan-PVA-PEG nanocomposites.

S. no.	Chitosan (grams)	PVA (grams)	PEG (grams)	Nanoclay (grams)	Drug (grams)	Glycerol (grams)
MA1	2.5	1.875	0.625	0	0	1.25
MA2	2.5	1.25	1.25	0	0	1.25
MA3	2.5	0.625	1.875	0	0	1.25
MA4	2.5	1.25	1.25	0.075	0	1.25
MA5	2.5	1.25	1.25	0.15	0	1.25
MA6	2.5	1.25	1.25	0.25	0	1.25
MA7	2.5	1.875	0.625	0.075	0.375	1.25
MA8	2.5	1.25	1.25	0.075	0.375	1.25
MA9	2.5	0.625	1.875	0.075	0.375	1.25
MA10	2.5	1.875	0.625	0	0.375	1.25
MA11	2.5	1.25	1.25	0	0.375	1.25
MA12	2.5	0.625	1.875	0	0.375	1.25

**Table 2:** Erosion data for CS-PVA-PEG formulations.

Formulation codes	Erosion (% ± SD)		
	pH 1.2	pH 4.5	pH 6.8
MA7	71.57 ± 0.08	63.50 ± 0.10	61.77 ± 0.09
MA8	72.25 ± 0.05	61.38 ± 0.10	59.16 ± 0.03
MA9	75.94 ± 0.13	68.67 ± 0.08	66.60 ± 0.13
MA10	74.51 ± 0.13	67.76 ± 0.08	66.50 ± 0.10
MA11	73.07 ± 0.16	65.40 ± 0.26	63.88 ± 0.16
MA12	73.82 ± 0.16	67.27 ± 0.13	65.52 ± 0.10

## Data Availability

The data used to support the results of this study are included within the supplementary information.

## Supplementary Materials

Supplementary Materials Table S1: FTIR table of chitosan-PVA-PEG composite films MA1–MA3. Table S2: FTIR table of chitosan-PVA-PEG nanocomposite films MA4–MA6. Table S3: FTIR table of chitosan-PVA-PEG nanocomposite films MA7–MA9. Table S4: FTIR table of chitosan-PVA-PEG composite films MA10–MA12. Table S5: TGA table of chitosan-PVA-PEG composite films MA1–MA3. Table S6: TGA table of chitosan-PVA-PEG nanocomposite films MA4–MA6. Table S7: TGA table of chitosan-PVA-PEG nanocomposite films MA7–MA9. Table S8: TGA table of chitosan-PVA-PEG composite films MA10–MA12. Table S9: XRD table of chitosan-PVA-PEG nanocomposite films MA1–MA12.
