# Supplementary material for: Nanoclay Reinforced Ternary Blends Based on Biodegradable Polymers for Drug Delivery Application
Source: Int J Biomater. 2022 Sep 7;2022:6585305. doi: 10.1155/2022/6585305 (PMC9473880; doi:10.1155/2022/6585305)
Supplement: Supplementary Materials — Table S1: FTIR table of chitosan-PVA-PEG composite films MA1–MA3. Table S2: FTIR table of chitosan-PVA-PEG nanocomposite films MA4–MA6. Table S3: FTIR table of chitosan-PVA-PEG nanocomposite films MA7–MA9. Table S4: FTIR table of chitosan-PVA-PEG composite films MA10–MA12. Table S5: TGA table of chitosan-PVA-PEG composite films MA1–MA3. Table S6: TGA table of chitosan-PVA-PEG nanocomposite films MA4–MA6. Table S7: TGA table of chitosan-PVA-PEG nanocomposite films MA7–MA9. Table S8: TGA table of chitosan-PVA-PEG composite films MA10–MA12. Table S9: XRD table of chitosan-PVA-PEG nanocomposite films MA1–MA12. [file 6585305.f1.zip › Table S1.doc]

**Table S1: FTIR table of Chitosan-PVA-PEG composite films MA1-MA3**

| **Chitosan** | **PVA** | **PEG** | **MA1** | **MA2** | **MA3** |
| --- | --- | --- | --- | --- | --- |
| **OH**  **(3334 cm^-1^)** | **3550-3200 cm^-1^** | **3441 cm^-1^** | 3322 cm^-1^ | 3296 cm^-1^ | 3292 cm^-1^ |
| **C=O Amide I (1648 cm^-1^)** | **----** | **----** | 1640 cm^-1^ | 1646 cm^-1^ | 1646 cm^-1^ |
| **N-H bending Amide II**  **(1594 cm^-1^)** | **----** | **----** | 1545 cm^-1^ | 1537 cm^-1^ | 1557 cm^-1^ |
