# Supplementary material for: Nanoclay Reinforced Ternary Blends Based on Biodegradable Polymers for Drug Delivery Application
Source: Int J Biomater. 2022 Sep 7;2022:6585305. doi: 10.1155/2022/6585305 (PMC9473880; doi:10.1155/2022/6585305)
Supplement: Supplementary Materials — Table S1: FTIR table of chitosan-PVA-PEG composite films MA1–MA3. Table S2: FTIR table of chitosan-PVA-PEG nanocomposite films MA4–MA6. Table S3: FTIR table of chitosan-PVA-PEG nanocomposite films MA7–MA9. Table S4: FTIR table of chitosan-PVA-PEG composite films MA10–MA12. Table S5: TGA table of chitosan-PVA-PEG composite films MA1–MA3. Table S6: TGA table of chitosan-PVA-PEG nanocomposite films MA4–MA6. Table S7: TGA table of chitosan-PVA-PEG nanocomposite films MA7–MA9. Table S8: TGA table of chitosan-PVA-PEG composite films MA10–MA12. Table S9: XRD table of chitosan-PVA-PEG nanocomposite films MA1–MA12. [file 6585305.f1.zip › Table S5.doc]

**Table S5: TGA table of Chitosan-PVA-PEG composite films MA1-MA3**

| **Sample code** | **% Weight loss at** | | | **% weight residue**  **540 °C** |
| --- | --- | --- | --- | --- |
|  | **132 °C** | **209 °C** | **404 °C** |  |
| **MA1** | 8.2 | 37.6 | 68.6 | 17.3 |
| **MA2** | 5.3 | 7.4 | 37.6 | 43.9 |
| **MA3** | 5.2 | 9.1 | 28.5 | 59.2 |
