# Supplementary material for: Nanoclay Reinforced Ternary Blends Based on Biodegradable Polymers for Drug Delivery Application
Source: Int J Biomater. 2022 Sep 7;2022:6585305. doi: 10.1155/2022/6585305 (PMC9473880; doi:10.1155/2022/6585305)
Supplement: Supplementary Materials — Table S1: FTIR table of chitosan-PVA-PEG composite films MA1–MA3. Table S2: FTIR table of chitosan-PVA-PEG nanocomposite films MA4–MA6. Table S3: FTIR table of chitosan-PVA-PEG nanocomposite films MA7–MA9. Table S4: FTIR table of chitosan-PVA-PEG composite films MA10–MA12. Table S5: TGA table of chitosan-PVA-PEG composite films MA1–MA3. Table S6: TGA table of chitosan-PVA-PEG nanocomposite films MA4–MA6. Table S7: TGA table of chitosan-PVA-PEG nanocomposite films MA7–MA9. Table S8: TGA table of chitosan-PVA-PEG composite films MA10–MA12. Table S9: XRD table of chitosan-PVA-PEG nanocomposite films MA1–MA12. [file 6585305.f1.zip › Table S6.doc]

**Table S6: TGA table of Chitosan-PVA-PEG nanocomposite films MA4-MA6**

| **Sample code** | **% Weight loss at** | | | **% weight residue** |
| --- | --- | --- | --- | --- |
|  | **132 °C** | **209 °C** | **327 °C** |  |
| **MA4** | 6.1 | 26.1 | 56.6 | 20.6 |
| **MA5** | 8.9 | 49.7 | 70.0 | 15.6 |
| **MA6** | 9.0 | 41.4 | 59.6 | 15.9 |
