# Supplementary material for: Nanoclay Reinforced Ternary Blends Based on Biodegradable Polymers for Drug Delivery Application
Source: Int J Biomater. 2022 Sep 7;2022:6585305. doi: 10.1155/2022/6585305 (PMC9473880; doi:10.1155/2022/6585305)
Supplement: Supplementary Materials — Table S1: FTIR table of chitosan-PVA-PEG composite films MA1–MA3. Table S2: FTIR table of chitosan-PVA-PEG nanocomposite films MA4–MA6. Table S3: FTIR table of chitosan-PVA-PEG nanocomposite films MA7–MA9. Table S4: FTIR table of chitosan-PVA-PEG composite films MA10–MA12. Table S5: TGA table of chitosan-PVA-PEG composite films MA1–MA3. Table S6: TGA table of chitosan-PVA-PEG nanocomposite films MA4–MA6. Table S7: TGA table of chitosan-PVA-PEG nanocomposite films MA7–MA9. Table S8: TGA table of chitosan-PVA-PEG composite films MA10–MA12. Table S9: XRD table of chitosan-PVA-PEG nanocomposite films MA1–MA12. [file 6585305.f1.zip › Table S8.doc]

**Table S8: TGA table of Chitosan-PVA-PEG composite films MA10-MA12**

| **Sample code** | **% Weight loss at** | | | **% weight residue** |
| --- | --- | --- | --- | --- |
|  | **111 °C** | **205 °C** | **340 °C** |  |
| **MA10** | 4.3 | 25.1 | 51.3 | 23.4 |
| **MA11** | 1.4 | 27.6 | 49.1 | 24.2 |
| **MA12** | 4.5 | 19.0 | 34.5 | 27.9 |
