# Supplementary material for: Nanoclay Reinforced Ternary Blends Based on Biodegradable Polymers for Drug Delivery Application
Source: Int J Biomater. 2022 Sep 7;2022:6585305. doi: 10.1155/2022/6585305 (PMC9473880; doi:10.1155/2022/6585305)
Supplement: Supplementary Materials — Table S1: FTIR table of chitosan-PVA-PEG composite films MA1–MA3. Table S2: FTIR table of chitosan-PVA-PEG nanocomposite films MA4–MA6. Table S3: FTIR table of chitosan-PVA-PEG nanocomposite films MA7–MA9. Table S4: FTIR table of chitosan-PVA-PEG composite films MA10–MA12. Table S5: TGA table of chitosan-PVA-PEG composite films MA1–MA3. Table S6: TGA table of chitosan-PVA-PEG nanocomposite films MA4–MA6. Table S7: TGA table of chitosan-PVA-PEG nanocomposite films MA7–MA9. Table S8: TGA table of chitosan-PVA-PEG composite films MA10–MA12. Table S9: XRD table of chitosan-PVA-PEG nanocomposite films MA1–MA12. [file 6585305.f1.zip › Table S9.doc]

**Table S9: XRD table of Chitosan-PVA-PEG nanocomposite films MA1-MA12**

| **MA1** | **2 theta** | **d-spacing** | **intensity** |
| --- | --- | --- | --- |
|  | 6.06 | 14.5694 | 557.48 |
|  | 12.18 | 7.2607 | 336.66 |
|  | 20.30 | 4.3718 | 2838.06 |
|  |  |  |  |
| **MA 2** | 6.26 | 14.1017 | 261.82 |
|  | 11.76 | 7.522 | 146.99 |
|  | 19.52 | 4.5439 | 1068.70 |
|  | 20.15 | 4.4029 | 1197.32 |
|  | 20.91 | 4.2451 | 1142.20 |
|  |  |  |  |
| **MA 3** | 9.83 | 8.9922 | 384.21 |
|  | 21.14 | 4.199 | 4126.33 |
|  | 23.36 | 3.8049 | 2831.59 |
|  |  |  |  |
| **MA 4** | 6.19 | 14.2727 | 565.64 |
|  | 10.43 | 8.4722 | 1060.57 |
|  | 20.19 | 4.3951 | 5522.06 |
|  |  |  |  |
| **MA 5** | 5.19 | 17.0066 | 502.93 |
|  | 11.53 | 7.6679 | 410.29 |
|  | 20.05 | 4.4257 | 5174.92 |
|  | 22.22 | 3.9968 | 4758.01 |
|  |  |  |  |
| **MA 6** | 5.13 | 17.2195 | 296.51 |
|  | 9.73 | 9.0818 | 226.82 |
|  | 19.26 | 4.6054 | 1016.61 |
|  | 19.96 | 4.4447 | 1067.17 |
|  | 21.56 | 4.1186 | 873.14 |
|  |  |  |  |
| **MA 7** | 6.06 | 14.5684 | 438.03 |
|  | 9.81 | 9.0094 | 374.32 |
|  | 12.68 | 6.9741 | 479.84 |
|  | 15.99 | 5.5372 | 617.22 |
|  | 17.93 | 4.9434 | 1192.63 |
|  | 19.62 | 4.5220 | 1560.98 |
|  | 21.11 | 4.2042 | 1429.57 |
|  | 22.99 | 3.8656 | 1262.32 |
|  |  |  |  |
| **MA 8** | 5.38 | 16.4076 | 383.70 |
|  | 11.74 | 7.5287 | 300.07 |
|  | 21.16 | 4.1949 | 3846.82 |
|  | 26.06 | 3.4163 | 1018.28 |
|  |  |  |  |
| **MA 9** | 6.26 | 14.0989 | 575.30 |
|  | 13.97 | 6.3327 | 327.32 |
|  | 20.10 | 4.4135 | 3833.66 |
|  | 21.62 | 4.1072 | 3878.30 |
|  | 23.14 | 3.8413 | 3570.81 |
|  | 26.36 | 3.3785 | 1324.18 |
|  |  |  |  |
| **MA 10** | 17.81 | 4.9761 | 355.50 |
|  | 19.58 | 4.5308 | 987.02 |
|  | 20.71 | 4.2848 | 929.22 |
|  | 22.67 | 3.9193 | 508.69 |
|  | 23.68 | 3.7544 | 390.18 |
|  |  |  |  |
| **MA 11** | 9.90 | 8.9244 | 465.19 |
|  | 21.97 | 4.0420 | 4502.57 |
|  | 25.18 | 3.5341 | 1619.55 |
|  |  |  |  |
| **MA 12** | 18.20 | 4.8703 | 518.29 |
|  | 19.16 | 4.6292 | 722.01 |
|  | 19.73 | 4.4959 | 1004.71 |
|  | 20.69 | 4.2901 | 953.44 |
|  | 22.15 | 4.0093 | 749.72 |
